# How to Modulate Peripheral and Central Nervous System to Treat Acute Postoperative Pain and Prevent Pain Persistence

**DOI:** 10.2174/1570159X21666230810103508

**Published:** 2023-08-15

**Authors:** Sara Cazzaniga, Giovanni Real, Simone Finazzi, Luca F. Lorini, Patrice Forget, Dario Bugada

**Affiliations:** 1 Emergency and Intensive Care Department, ASST Papa Giovanni XXIII, 24127, Bergamo, Italy;; 2 Department of Health Sciences, University of Milan, 20122, Milan, Italy;; 3 School of Medicine, Medical Sciences and Nutrition, Epidemiology Group, Institute of Applied Health Sciences, University of Aberdeen, Scotland, United Kingdom;; 4 Department of Anaesthesia, NHS Grampian, Aberdeen AB25 2ZD, Scotland, United Kingdom

**Keywords:** Chronic postoperative pain, persistent pain, postoperative pain, hyperalgesia, regional anesthesia, opioid free anesthesia, adjuvants

## Abstract

Chronic postoperative pain (CPSP) is a major issue after surgery, which may impact on patient’s quality of life. Traditionally, CPSP is believed to rely on maladaptive hyperalgesia and risk factors have been identified that predispose to CPSP, including acute postoperative pain. Despite new models of prediction are emerging, acute pain is still a modifiable factor that can be challenged with perioperative analgesic strategies. In this review we present the issue of CPSP, focusing on molecular mechanism underlying the development of acute and chronic hyperalgesia. Also, we focus on how perioperative strategies can impact directly or indirectly (by reducing postoperative pain intensity) on the development of CPSP.

## 1 INTRODUCTION

Chronic post-surgical pain (CPSP) is one of the most serious, long-term consequences of surgery. CPSP is a condition that often affects adult patients who receive surgery depending on the type of procedure. CPSP has been recently classified as a specific type of chronic pain by IASP (International Association for the Study of Pain). It is defined by three main characteristics: 1) the pain is caused by a surgical procedure, 2) the pain persists beyond the healing process (time range: 3 months after surgery) and 3) other causes for pain as a continuation of a pre-existing problem, chronic infection or recurring of malignancy must be excluded [1]. Basically, CPSP is a pain that develops as a “new” pain or exacerbation of existing pain after surgery, involving the surgical site or nearby territory, and lasts for longer than expected, probably because of some abnormalities in the pain pathway created or exacerbated by surgery.

As in other diseases, options for either primary or secondary prevention theoretically exist. Primary prevention would mean not having surgery at all, but that is not an option in most cases. Secondary prevention includes early detection and intervention and is most applicable to patients who undergo surgery. This form of prevention can take place only by understanding the mechanisms of CPSP, and the underlying risk factors and by following up with the patients in the delicate period of the transition from acute postoperative pain towards rehabilitation and resumption of normal daily life (hopefully, without CPSP).

In this context, perioperative pain is recognized as an important risk factor and a potential target for secondary prevention [2]. In this article, we want to summarize the available evidence on CPSP epidemiology, pathophysiology, and risk factors. Further, we will focalize on the role of postoperative pain (POP) as a potentially preventable risk factor for CPSP, with special attention on the early pharmacologic approaches that may help reduce pain transition to chronic disease.

## 2 CHRONIC POSTOPERATIVE PAIN: EPIDEMIOLOGY, MECHANISMS, AND IMPACT

### 2.1 The Magnitude of the Problem

The first robust epidemiological reports about the impact of CPSP date back to the nineties [3]. Nowadays, CPSP is a worldwide medical, social, and economic problem [4, 5].

CPSP has not been homogeneously defined, and different authors have often considered different criteria for the detection of CPSP [6, 7]. However, CPSP incidence has not changed significantly over years (Table **1**) [3, 6, 8-10], ranging from 5% to 85% (considering different surgical techniques and types of surgery) [5]. This interval includes moderate, mild, and severe CPSP (the latter greatly affecting the patient’s quality of life, with an estimated incidence of 5-10%) [6]. The lack of a clear definition is the cause of the wide range of incidences. At the very beginning, any pain 2 months post-surgery was considered CPSP. In 2014, a re-definition of CPSP was proposed that also considered the impact of sensory abnormalities and pain rather than their occurrence itself. Currently, CPSP includes any pain significantly affecting the quality of life, specifically pain 3-6 months after surgery when other causes for pain are excluded in the area of surgery or projected to the innervation territory of a nerve situated in the surgical field, or referred to a dermatome (after surgery in deep somatic or visceral tissues). CPSP includes the pain of develops after a surgical procedure or increases in intensity after the surgical procedure, either a continuation of acute post-surgery pain or develops after an asymptomatic period [3].

In any case, the problem of CPSP from a healthcare perspective is mainly related to the absolute impact rather than to the relative number of patients affected in each surgery. Despite having a low rate of occurrence in some surgeries, the healthcare impact is high given the total number of surgical procedures performed every year. CPSP negatively affects the quality of life [11], leading to disability and being often refractory to treatment; CPSP is also shown to be one of the underlying causes of opioid use disorders [7]. Therefore, there is great interest in developing preventative strategies to decrease the development of CPSP.

### 2.2 Risk Factors for Chronic Postoperative Pain

Chronic postoperative pain is a complex phenomenon. Each individual reacts to surgical insults uniquely [6]. CPSP is believed to be an abnormal consequence of acute pain and the main mechanism underlying the development of CPSP is believed to be, at least partly, mediated by a dysregulation in the mechanism of hyperalgesia. This might be linked to different underlying conditions and factors.

Several risk factors have been identified over years (Table **2**). Most of them are un-modifiable (like genetics, sex, gender, preoperative medical conditions including pain or chronic opioid therapy), but still worth to be considered to identify patients at risk (at least) of experiencing higher postoperative pain (the most important risk factor for CPSP - see below). As some factors cannot be influenced, acute pain does: effective POP management may reduce the influence of this (at least partially) preventable risk factor for pain persistence.

Pain management after surgery is significant to determine CPSP. Historically, acute pain has been associated with CPSP occurrence. Considering the complexity of pain, the concept and the association between acute pain and CPSP has been recently challenged: “severity” but not “presence” of acute pain is likely linked to CPSP (as indicative of individual abnormal pain processing). The intensity of acute preoperative pain correlates with the onset of chronic pain, particularly during mobilization [12]: a recent study has concluded that a 10% increase in the percentage of time in severe pain was associated with a 30% increase in CPSP incidence at 12 months [5].

For years, the concept has been that “the greater the surgery, the higher the pain”: surgery has been the first risk factor for CPSP to be investigated. CPSP is highly associated with major procedures that are associated with a long duration of surgery, extensive tissue manipulation, and the high incidence of direct nerve damage, as well as multiple interventions or surgery in a previously injured area. However, minor surgeries are not free from this phenomenon. Recently, the incidence of PPSP after surgery by thoracoscopy has been documented to be the 25% in the best case, and even small procedures like hernia repair are associated with persistent pain [13-15]. On this basis, the paradigm is shifting from a surgery-oriented model (where the degree of surgical insult is the main determinant of CPSP) to a patient-oriented approach: the same surgical procedure can generate different responses in different individuals and develop different degrees of CPSP [16]. To explain this variability, a biopsychosocial model was developed [17], based not only on surgery but also on patients’ characteristics. Uncontrolled acute pain biologically predisposes to chronic pain by altering neuroendocrine patterns but also decompensating the psychological status [18].

Neuropathic pain can be caused by a disfunction or a lesion either of the central or peripheral nervous system and can be associated with direct nerve lesions or central sensitization [19]. Neuropathic pain has a great role in the development of CPSP: regardless of invasiveness or the extent of surgical manipulation, the degree of nerve lesion seems to be a major determinant for CPSP. Haroutiunian *et al.,* [19] showed that the incidence of neuropathic pain after surgery widely varies (also according to the methodologic variability in detecting neuropathic pain among studies), but surgeries with a higher risk of developing neuropathic pain are those with a higher risk of iatrogenic nerve injury [19]. Moreover, a neuropathic component of pain can develop earlier in the perioperative period and may account for neuropathic CPSP: observational data on a wide cohort of surgical patients showed that neuropathic pain on the day of surgery or day 2 after surgery was a significant risk factor for neuropathic CPSP (with a remarkable 4.22 odds ratio) [20]. Some minor surgeries have a higher risk of direct nerve lesions, while some major surgeries do not have at same extent: this may explain (at least in part) why some minor interventions are still associated with the incidence of CPSP.

Quantitative sensory testing (QST) may be of great interest as a predictive tool to identify patients with a higher risk of CPSP or developing sensory abnormalities predisposing to CPSP. QST refers to psychophysical methods used to quantify somatosensory functioning; QST has been used on patients with a variety of pain conditions to test sensory profiles or subgroups. However, recent data show that no particular QST profile is unique to a specific diagnosis and that painful and pain-free neuropathies express similar QST [21]. This is of special interest: as different neuropathies are not distinguishable based on QST, different patterns of sensory abnormalities may reflect unique pain mechanisms [22]. QST phenotype may be valuable for specific therapeutic approaches, as well as to identify patients with underlying neuropathic conditions (even when clinically silent). In this perspective, QST has already been applied in predictive contexts. Pre-surgical individual differences in sensory profiles have shown prospective associations with acute and chronic post-operative pain across many procedures [23, 24]. A recent systematic review demonstrates that QST predicts CPSP and analgesic effects. However, the heterogeneity in methodologies reduces the generalizability and calls for methodological guidelines [25]. Further, QST can be time- and resource- consuming (difficult to manage each patient on a daily clinical base). However, a brief “bedside” QST (conveniently performed in a half hour or less) has been recently validated and is promising for future clinical applications [26].

Other preoperative factors may account for the development of CPSP that need to be integrated with the model. A role in CPSP insurgence has been attributed to opioid use. Either opioid assumption before surgery or administration during surgery is associated with opioid-induced hyperalgesia. Opioids amplify neuronal mechanisms of pain but also triggers inflammatory response [27]. From a clinical perspective, patients with long-term pain and chronic use of opioid analgesics before surgery are challenging. Some of them may have already developed some degree of sensitization and hyperalgesia before surgery [28], or tolerance/dependence/abuse to opioid medications. Sensitized and tolerant patients will be therefore expecting to experience higher levels of pain or to be more resistant to some drugs. Also, they may be exposed to prejudice by care providers, which possibly leads to the under treatment of pain in the perioperative setting [29]. Finally, difficulties in the adaptation of chronic therapies in the perioperative setting are a further factor that increases the incidence of ineffective pain management, with the risk of these specific patient populations experiencing severe pain after surgery [29].

Other preoperative conditions have been identified that help recognizes patients with a higher risk of perioperative pain. All conditions that are associated with a generalized inflammatory state are considered risk factors for CPSP. As inflammation is a major mechanism underlying pain and hyperalgesia in the nervous system, the systemic inflammatory state has been linked to chronic pain after surgery and the worst surgical outcomes in some studies. Patients with altered (pro-) inflammatory state before surgery or with documented pre-existing medical conditions associated with altered inflammatory balance displayed the worst functional outcomes and quality of life after joint replacement months or years after surgery [30, 31]. Rheumatologic diseases, autoimmune diseases, irritable bowel syndrome, and chronic inflammatory bowel diseases have been advocated among risk factors. In addition, obesity may play a major role as a cause of a pro-inflammatory state. Obesity induces a low-grade systemic inflammatory state characterized by the production and secretion of several adipocytokines that may have a role in osteoarthritis development. Moreover, local adipose tissue (like the infrapatellar fat pad in the knee) is a local source of cytokines and potentially contributes to osteoarthritis pathogenesis. Furthermore, hypertension, impaired glucose, and lipid metabolism, which are comorbidities associated with obesity, have been shown to alter joint tissue homeostasis. Fat itself (to be considered as an immune compound) releases specific cytokines involved as neuroinflammatory mediators that may therefore contribute to local and systemic inflammation and predispose to pain, even if a reactive inflammatory response may be, in some cases, protective as well [32-34].

Other factors that exist are associated with both postoperative and chronic pain. Among them are gender, age, and physiologic status (Table **2**).

Men have less pain sensitivity and reduced incidence of CPSP than women. Hormonal influence might be a reason however, clinical data are limited [35, 36]. Advanced age seems to be protective of CPSP occurrence. Even if elderly patients often suffer from chronic pain conditions they report lower pain intensity after surgery than younger patients. Indeed, younger females with greater pain sensitivity or preoperative uncontrolled pain (*e.g*. fibromyalgia patients) are more affected by CPSP.

Finally, in the context of the bio-psycho-social model, a greater risk factor for CPSP is a medical history of anxiety, depression, sleep disturbance, and catastrophizing status. Preoperative anxiety, fear of surgery, pain, and outcomes of surgery, as well as the tendency to exaggerate pessimism towards life (in general) and surgery (in the specific case), are all part of a negative vision of life. These patients are convinced that they will not be able to face surgery and pain, that they will not have any support from their family network and care practitioner, and that they will invariably experience some issues (pain especially) after surgery. These patients expect pain, they fear pain, and their negative expectation can be a predisposing factor to CPSP more than the actual pain experienced in the postoperative period [37].

A possible explanation is the so-called hypervigilance phenomenon [38]. In some patients, CPSP may be part of a hypervigilant status unmasked by surgery, in a vulnerable population [2]. Deregulation of the discrimination process (which has a major role in pain) was incriminated in some medical conditions like fibromyalgia: patients show amplification of all the sensory modalities, including pain, and share some of the risk factors of developing important acute postoperative pain and CPSP (female gender, anxiety, catastrophizing). Often, a history of trauma (like surgery) is reported as a precipitating factor of the disease [39]. Fragility (defined as socio-economic status, medical care access, and family network) is also worth to be mentioned. Wealth, good medical insurances, and strong networks positively impact pain severity: all these factors are strictly interconnected and act in a synergic fashion.

Most of the above-mentioned factors have been combined in a preoperative assessment score to identify patients at risk of severe POP, called the Kalkman score [40, 41]. Once combined, a sum > 4 identifies patients suitable to receive aggressive postoperative analgesia because at higher risk of intense postoperative pain (Table **2**) [42-48].

### 2.3 New Models for CPSP Prediction and Patient Stratification

Traditional prediction models are based on preoperative, static risk factors to identify the patients prone to a future outcome. The same models have been used for CPSP prevention, *i.e*. to identify patients at risk for CPSP. In other words, we traditionally aim for pre-determined phenotypes that need to be highly controlled along the perioperative course for their pre-disposition to CPSP.

CPSP is way more complex than that (as shown by our incomplete understanding of the topic). CPSP develops in a complex system where nervous structures interact with, genetic, humoral, and psychologic components in a dynamic fashion [49]. Pain itself (as well as drugs) may differently interact with the system, by changing his pre-operative, basal status to a new one. Any new variable to the system may add further changings in the status, providing the base for different modifications in pain processing in different patients.

In this regard, a different approach is required [50]. Mathematics and statistic may help in this regard, as they can process a huge amount of data to create adaptable models that consider how surgical pain and perioperative treatment impact pain pathways in different patients [51]. These models may overcome the limitations of the existing approaches, which are focused on the expected consequences of traditional preoperative risk factors [52].

The future of CPSP understanding may lie in machine-learning, algorithms that can autonomously process and integrate complex datasets. These algorithms are widely used and their application to medicine has recently been considered, also for postoperative pain prediction and CPSP [53, 54].

Complex algorithms are also changing the concept of transitional models. New studies have identified the so-called “sub-acute” period (first 2-4 weeks after surgery) as a more sensitive window to identify patients that are not resolving their pain or that display specific signs of abnormal adaptation of pain perception [54]. These “red flags” integrate the common preoperative risk factors, but can only be identified with a dynamic approach to the concept of CPSP. Theoretically, Artificial Intelligence could help in the clinical setting in the near future, estimating how specific techniques may protect patients in the context of high-risk procedures.

## 3 METHODS

As this is a narrative review, we did not follow strict recommendations like systematic review and metanalysis. PubMed and MEDLINE databases were searched until July 2022 using the combination of the following keywords: “chronic pain”, “postoperative pain”, “postsurgical pain”, “postoperative pain treatment”, “quantitative sensory testing”, “neuropathic pain”, “inflammation”, “central inflammation”, “descending inhibitory pathways”, “nociception”, “sodium channels”, “c-fos”, “ectopia”, “serotoninergic pathways”, “rostro ventromedial medulla”, “chemokines”, “endocannabinoid system”, “ketamine”, “non-steroidal anti-inflammatory drugs”, “paracetamol”, “acetaminophen”, “clonidine”, “dexmedetomidine”, “alpha-two agonists”, “magnesium”, “regional anesthesia”, “epidural analgesia”, “paravertebral block”, peripheral nerve blocks”, “opioids”, “gabapentinoids”.

All relevant papers were considered, with no restrictions on RCTs or metanalyses. Only papers in English were considered. Additional articles were identified from reference lists of retrieved papers.

## 4 MECHANISM UNDERLYING CPSP

### 4.1 Pathophysiology of CPSP – the Concept of Physiologic and Pathologic Hyperalgesia

The surgical insult activates primary and secondary hyperalgesia, which are physiological responses to acute pain in any case of injury in the human body.

Primary and secondary hyperalgesia differ in the site of action. Primary hyperalgesia occurs in the area supplied by a damaged nerve or in an inflamed area, whereas, secondary hyperalgesia occurs outside the area primarily interested by injury. According to another definition, we differentiate primary and secondary hyperalgesia because the former affects primary afferent nociceptors (also called peripheral sensitization) while the latter affects the central nervous system (CNS) (central sensitization) [55].

Acute (time-limited) hyperalgesia is a physiological phenomenon after surgery. In some cases, hyperalgesia is not time-limited: pain and inflammation last over time, and chronic hyperalgesia develops and lasts longer than the painful stimuli and the healing process. If as acute hyperalgesia always occurs, chronic hyperalgesia is a maladaptive phenomenon that only affects some patients, believed to be the main mechanism underlying the development of CPSP. Thus, CPSP is considered a maladaptive condition associated with a dysfunctional nociceptive system, that creates pain regardless of the presence (or not) of a painful stimulus.

Many physiological modifications sensitize the system and maintain this status. Pain as a sensory modality implicates both perceptions (to acknowledge that something happens) and discrimination (the ability to understand that what is happening now is different from before). Different mechanisms and structures exist along the pain pathways that account for the transduction of the chemical or mechanical stimulus in an electric current and transmission of the signal through nerve structure to the CNS (where discrimination happens and creates the cognitive elaboration of the stimulus, including the subjective experience associated with pain). All along the circuit, modulation of this perception exists: pain perception can be modified in a bidirectional manner, with mechanisms amplifying (sensitization) and others (descending inhibition) reducing pain. Normally, these mechanisms are in balance and may display transient adaptation when a pain stimulus occurs. A loss of balance is part of the maladaptive condition leading to CPSP.

Three major factors must be considered in this regard: (1) Increased primary discharge, (2) Altered inhibitory descending modulation, and (3) modulation by non-neural cells.

### 4.2 Increased Primary Discharge

The first response to a surgical insult is primary hyperalgesia that originates from sensitization of the primary afferent in the periphery (peripheral sensitization): pro-inflammatory cytokines are produced in the injured area and nearby, where as, injured nerves produce inflammatory molecules (neurogenic inflammation). All these mediators attract other inflammatory cells and perpetuate the process; the nociceptors are finally immersed in the so-called “inflammatory soup”, which has the effect of lowering the discharge threshold of nociceptors, facilitating the transduction of the external stimulus into an electric current [56]. Once the surgical stimulus comes, the level of neurotransmitters is quickly altered and structural changes occur within the cell bodies and in the central terminals of these afferent neurons [56].

In case of nerve injury, electric activity can also originate from places that do not normally generate electrical impulses (primary afferent neuronal cell bodies located in the dorsal root ganglion), it is referred to as “ectopic activity”. Ectopic activity originates days after nerve injury and can persist for several months. It is believed to rely on spontaneous discharge due to altered voltage-gated ion channel transport and expression after nerve injury and demyelinization [57].

The sensitization process naturally spreads to the level of the central nervous system [58] (“central sensitization”), which implies secondary hyperalgesia (expansion of the painful area outside the site of injury). Clinical manifestations of hyperalgesia are exacerbated pain in response to noxious stimulation and allodynia which is pain perceived to normally non-noxious stimuli.

### 4.3 Descending Modulation

The mechanisms by which the supraspinal centers can modulate the activity of neurons in the dorsal horn are complex. The descending serotonin pathway, which originates in the ventromedial rostral medulla (RVM) of the brain, is revealed to play a major role.

RVM is composed of “on cells” that accelerate their discharges and “off cells” which slow down their activity after a prolonged noxious stimulus. The normal sensitizing activity of RVM is increased in pathological conditions (facilitatory effect on pain), such as in the case of experimental models for peripheral nerve injury, inflammatory pain, and intense noxious stimulation [59, 60]. As a matter of fact, silencing the RVM after sciatic nerve ligation reduces pain hypersensitivity in rats [61]. In general, the RVM seems involved in the maintenance of this pathological condition, rather than in the initial input. Specific deletion of on-cells in the RVM was found to leave the onset of mechanical hypersensitivity unaffected, but the maintenance of mechanical hypersensitivity beyond the first week was lost [62-64]. RVM activation appears to come from NK-1 nuclei expressing the receptor for substance P [65], thus suggesting the relevance of spinal-supraspinal serotonergic loops [66].

On the other hand, inhibition of serotonin reuptake can reduce pain. Selective depletion of serotonergic innervations from RVM to the spinal cord was found to reduce gasp behavior in the formalin test (an experimental model of intense noxious stimulation), attenuation of mechanical hypersensitivity in the CFA (Complete Freund’s Adjuvant) model of inflammatory pain, and also reduced pain hypersensitivity after sciatic nerve ligation [66]. As well, antidepressant medication (serotonin reuptake inhibitors) can reduce pain-related symptoms by acting on the RVM in patients with chemotherapy-induced neuropathy [67]. Finally, in the SMIR (skin/muscle incision and retraction) model, experimental injury creates bilateral or unilateral mechanical allodynia, with increased levels of serotonin and its type 3 receptor in both bilateral and unilateral wounds [68].

However, supra-spinal centers do not play a facilitating role in pain. The noradrenergic pathways have instead an inhibitory role to the ascending nociceptive discharge, the locus coeruleus (LC) modulates (inhibition) the spinal nociceptive neurons by inhibiting the release of norepinephrine. The down-regulation of LC neurons has been reported in neuropathic models [66], while recent findings suggest a relationship between LC activity and neural inflammation. The selective activation of the LC-spinal cord pathway alleviates neuropathic pain in mice by reducing neuroinflammation of astrocytes and microglia in the dorsal horn [69]. On the other hand, elevated neuroinflammation and microglial activation in the brain and spinal cord of mice correlate with significant degeneration of the LC-norepinephrine system [70]. The role of LC is not restricted to pain in the central nervous system, with a wide range of implications that are common features of patients with chronic pain. In patients with chronic pain, LC activity produces pain facilitation, anxiety, increased aversive memory, and behavioral despair, acting at the medulla, prefrontal cortex, and amygdala levels. The LC displays a central role in chronic pain states, and the activation/deactivation of specific LC projections contributes to behavioral outcomes (rather than only pain inhibition) that are central items in the bio-psychosocial model of CPSP [71].

### 4.4 Modulation by Non-neural Cells

Activation of non-neural cells and neuro-glial interactions are emerging as key mechanisms underlying pain. These cells are the main immune-competent cells within the central and peripheral nervous system and act as mediators of inflammatory and immune activation following a painful stimulus.

Microglia are macrophage-like cells in the CNS that originate from bone marrow-derived monocytes, distributed throughout the CNS. They sense their environment and interact with synapses to modulate their structure and functions. Astrocytes are the most abundant cells in the CNS and were historically regarded as support cells. A recent “tripartite synapse” theory (where glia responds to neuronal activity and triggers the release of chemical transmitters that cause feedback regulation of neuronal activity) proposes astrocytic processes as an active component of synapses, in addition to pre- and post-synaptic components [72]. This theory is hardly debated, but alternative pathways for astrocytic modulation of synaptic transmission have been proposed in the maintenance of potassium hemostasis: extracellular concentration of potassium is important for resting membrane potential and neuronal activity [73].

Glial cells also exist in the peripheral nervous system: they are satellite glial cells (SGCs) in the dorsal root ganglia and trigeminal ganglia and Schwann cells in the peripheral nerves. SGCs are characterized by thin cellular sheaths that surround the individual neurons, with similarities to astrocytes. Emerging evidence suggests that SGCs are activated by painful injuries and play an active role in the development of persistent pain [74-78]. SGCs also exhibit enhanced coupling in persistent inflammatory and neuropathic pain [75-79].

Painful syndromes are associated with different glial activation states, which finally brings to the production of inflammatory mediators. Briefly, after glial reaction/priming (*i.e*., upregulation of glial markers and/or morphological changes, including hypertrophy, proliferation, and modifications of glial networks) an activation of cellular signaling pathways, transporters, and receptors takes place that finally leads to the synthesis and release of glial mediators to the extracellular space [80]. Glial activation brings to the production of inflammatory mediators including proinflammatory cytokines, but also chemokines, lipid mediators (*e.g*. prostaglandins), and growth factors which have a substantial impact on neuronal activity. Glial mediators powerfully modulate excitatory and inhibitory synaptic transmission at presynaptic, postsynaptic, and extra-synaptic sites [80]. Inflammatory mediators may either directly evoke neuronal activity or modulate it *via* disinhibition (impairment of inhibitory interneurons) or potentiation of excitatory neurotransmission. In any case, inflammation within the CNS put the system in a hyper-reactive state of pain perception [43]. However, glial priming is not thought to mediate pain sensitivity directly. Instead, glial activation finally enhances pain sensitivity *via* a number of synergistic neuro-glial interactions. Glial activation also occurs in acute pain conditions, but chronic pain could be a result of a “gliopathy,” *i.e*. a dysregulated of the glial functions nervous system [80].

## 5 PHARMACOLOGIC APPROACHES TO ACUTE PAIN TO PREVENT CPSP

The intensity and duration of acute pain play a major role in CPSP. Effective strategies for POP management have been therefore regarded as a potential target to prevent pain persistence.

In the last decades, the concept of multimodal analgesia has been validated, *i.e*. to combine analgesic strategies focused on the mechanism involved in pain pathways to improve analgesia while reducing side effects of treatment [81]. This concept mainly focuses on the mechanisms underlying pain rather than the sole intensity of pain. This approach is now moving to the intraoperative period (multimodal anesthesia) to influence pain perception from the earlier phase of the surgical insult and fight all sources of hyperalgesia from the very beginning, improving POP management and theoretically preventing from CPSP (preventive anesthesia and analgesia). In this regard, different strategies have been investigated, in constant research for a balance between efficacy, side effects, and feasibility in the context of everyday clinical pathways.

### 5.1 Non-steroidal Anti-inflammatory Drugs (NSAIDs)

NSAIDs should be considered for the perioperative pain management of all patients since they reduce opioid requirements and related adverse events while reducing recovery times in the PACU and morbidity [82]. Most of the drugs available are either reversible inhibitors of both cyclooxygenase (COX)-1 and COX-2 or selective inhibitors of COX-2 [83]. Even though no significant difference in effect has been proven, COX-2 inhibitors have fewer short-term adverse effects [84]. Historically, it was widely understood that at least part of the anti-inflammatory effect of NSAIDs was exerted thanks to their peripheral inhibition of COXs and other pro-inflammatory cytokines [85]. More recently convincing evidence has emerged in both animal and human studies showing that NSAIDs have also an important central mechanism of action [86]. In rat pain models, intrathecal delivery of NSAIDs reduced the nociceptive behavior even at dosages at which systemic action was negligible [87]. Some studies also proved that intrathecal injection of NSAIDs significantly reduced pain associated with an inflammatory stimulus in the long term [88]. According to the ASA (American Society of Anesthesiologists), all patients should undergo an NSAIDs/Acetaminophen combination in the postoperative period if no contraindications are present [89]. A recent Cochrane meta-analysis including 71 RCT, concluded that even when only administered before the surgery, NSAIDs are effective in reducing pain and opioid consumption [90]. Moreover, in a patient with no history of renal dysfunction or cardiac comorbidities, the perceived risk of renal dysfunction, bleeding, or gastrointestinal complications related to NSAIDs has been proven not to be of clinical importance [91]. There are initial evidence that suggest that NSAIDs may also play a role in the reduction of chronic hyperalgesia [92]. Evidence on the matter is still limited, and even though there are studies that seem to show no clear benefit of COX-2 inhibition to prevent chronic hyperalgesia [93], NSAIDs are starting to emerge as a valid tool in the context of a multimodal strategy to prevent the chronic pain [94], at least for their efficacy against POP.

### 5.2 N-Methyl-D-Aspartate (NMDA) Receptor Antagonism - Ketamine, Dextromethorphan and Magnesium

Even though a large number of NMDA receptor antagonists are commercially available, ketamine is the only one which is widely used in surgical patients. Ketamine binds a hydrophobic domain on the NMDA receptor which decreases the frequency of channel opening in case of stimulation [95]. Consequently, the influx of calcium ions through the channel is greatly reduced [96]. Ketamine also exerts its effect through the binding of the μ, κ, and δ opioids receptors [97]. However, since naloxone has been shown to have no effect on the analgesic properties of ketamine, its importance has been debated [98]. Part of ketamine is also an agonist of α and β adrenergic receptors [99], which also explains the hyperadrenergic state caused by ketamine that manifests with increased heart rate, blood pressure, and cardiac output. One of the most important side effects of ketamine is increased bronchial secretion due to a direct effect on muscarinic receptors [100].

Noteworthy, ketamine plays an important role in the modulation of both the immune system and the inflammatory response. The anti-inflammatory effect is more potent when ketamine is administered pre-emptively. Ketamine reduces the secretion of pro-inflammatory cytokines, mainly IL-6 and TNF-alpha, but it does so in a context-dependent manner. The greater the pro-inflammatory stimulus, the more potent the anti-inflammatory effect of ketamine; if the inflammatory stimulus is not present, ketamine does not alter the function of the immune cells [101]. These results are evident in preclinical and clinical studies; a meta-analysis involving more than 600 patients shows that intraoperative ketamine significantly reduces IL-6 in the postoperative period [102]. The levels of inhibition of post-operative IL-6 in patients who were administered sub-anesthetic doses of ketamine (0.15 mg x kg^-1^), were comparable to the ones achieved using methylprednisolone and the effect was significantly longer [102, 103].

As already mentioned, ketamine also modulates immune function. In the case of a pro-inflammatory stimulus, ketamine significantly increases the Th2/Th1 ratio. This shift in the lymphocyte population helps to increase the anti-inflammatory response, which is Th2 mediated and lowers the Th1-mediated pro-inflammatory response [104]. This immune-related effect is of particular interest, especially when compared to the one of morphine that greatly reduces the Th2/Th1 ratio [105, 106], with a consequent enhancement of pro-inflammatory cytokines.

The role of ketamine in preventing CPSP incidence is still debated [107]. However, current guidelines suggest that in the perioperative setting, sub-anesthetic doses of ketamine (0.35 mg x kg^-1^) [108] “should be considered for patients undergoing painful surgery” which includes upper abdominal and thoracic surgery, intra-abdominal surgery and orthopedic procedures [107]. Indeed, patients for which ketamine should be considered of special interest are opioid-tolerant patients and patients with important pre-operative painful states [107]. A recent RCT shows that intraoperative ketamine may reduce opioid use and pain and improve labour market attachment one year after spine surgery in an opioid-dependent patient [109].

Dextromethorphan is a morphine derivate that, despite its structural similarity to opioid agonists, has no direct action on opioid receptors [110]. It exerts its action thanks to interaction with various binding sites such as serotonin transporters, noradrenaline transporters, nicotinic receptors, and NMDA receptors [111]. The most recent meta-analysis on the use of dextromethorphan was published in 2016 and it included 21 RCTs. Despite a high degree of heterogeneity among the studies, perioperative administration of dextromethorphan significantly reduced post-operative pain scores up to 24h post-surgery [112].

Magnesium (Mg^2+^) is one of the most important physiological antagonists of the NMDA receptor, its analgesic properties derive from its blockage of the NMDA receptor at the level of the spinal cord. Moreover, the activation of the nitric oxide pathway which follows the administration of magnesium sulfate is thought to play a role in the analgesic properties of magnesium on somatic pain [113] A review published in 2021, shows that Mg^2+^ reduces pain scores when compared to control groups [114].

### 5.3 Alpha-2 Agonists

The two most important drugs belonging to the class of alpha-2 agonists are clonidine and dexmedetomidine. They reduce the sympathetic activity by reducing the norepinephrine release thanks to their binding with the pre-junctional alpha-2 receptor [115]. Other than their anti-hypertensive effect, they also act as analgesic, anxiolytics, and sedatives [116]. Results on clonidine have been reproduced for dexmedetomidine [117]. Of the two drugs, dexmedetomidine shows an earlier and stronger analgesic effect, which can be assessed with a greater morphine-sparing effect and a more evident reduction on the pain scale [118]. Two more recent meta-analyses however have shown promising results in the use of alpha-2 agonists as part of an effective multimodal analgesic plan for the management of acute post-operatory pain, also as adjuvants of regional anesthesia [119, 120]. Intrathecal clonidine has a postoperative anti-hyperalgesic effect, reducing the extent and incidence of peri-incisional punctate mechanical hyperalgesia after surgery [121]. At the spinal level, these effects are combined with the blunting effect on the surgical stress response [122] (mediated by pre-ganglionic block in the sympathetic chain).

### 5.4 Opioids

The analgesic effect of opioids is mainly due to their binding with the μ receptors in the central nervous system. Other opioid receptors such as the κ and δ are also bound to a lesser degree, but physiological effects are various and not necessarily analgesic [123]. Despite their numerous side effects such as somnolence, dizziness, constipation, nausea and vomiting, and respiratory depression, opioids remain one of the most powerful tools anesthesiologists have against acute intraoperative and postoperative pain. Since opioid-related adverse effects like nausea and vomiting and respiratory depression could lead to complications and prolonged recovery times, an ever increasing line of research has developed to explore the possibility of opioid-sparing and opioid-free anesthesia [124, 125]. In the most recent enhanced recovery after surgery (ERAS) analgesia protocols, opioids are still regarded as a fundamental drug class; however physicians are encouraged to adopt at least opioid-sparing protocols [126], and there is also evidence that even multimodal opioid-free regiments are at least as good as opioid-based regiments in providing analgesia intra and post-operatively [124, 127].

### 5.5 Gabapentinoids

Gabapentin and pregabalin both belong to the class of gabapentinoids, their pharmacological action in due to the binding with the α 2-delta subunit of the voltage-gated calcium channels. The consequence is the reduction of the release of various neurotransmitters such as glutamate, noradrenaline, and the substance P [128]. Gabapentinoids are mainly used as anti-convulsant or in the treatment of neuropathic pain. However, an ever-growing number of studies focusing on their potential use in the treatment of postoperative pain have been published [129, 130].

The evidence regarding their efficacy on acute post-operative pain is hardly debated. PROSPECT guidelines for oncologic breast surgery suggest that for all patients without contraindications “Pre‐operative gabapentin is recommended” [47]. However, the evidence of their efficacy in other surgical scenarios is concerning, especially due to the poor methodology of trials supporting their use and the frequent occurrence of undesirable side effects (like sedation and dizziness). Further,gabapentinoids are still not proven to significantly reduce the incidence of chronic post-operative neuropathic pain [131].

### 5.6 Regional Anesthesia (RA)

With the term “regional anesthesia” we usually refer to various procedures which involve the injection of local anesthetics near a nervous structure; examples are spinal or epidural anesthesia, where the drug is injected in the subarachnoid or epidural space respectively, or peripheral nerve blocks where a local anesthetic is injected directly next to a nerve plexus or a peripheral nerve. Local anesthetics are a relatively homogeneous class of drugs for structure and pharmacological targets. The most used in clinical practice are amides like lidocaine, ropivacaine, bupivacaine, and levobupivacaine, which inhibit neural transmission thanks to their binding with the voltage-dependent sodium (Na) channels [132]. A number of studies have proved that local anesthetics also play an important role in the modulation of the inflammatory response [133]. It has also been proved that lidocaine can inhibit the adhesion of granulocytes to the endothelium in a dose-dependent manner [134]. Adhesion, however, is not the only step of the inflammatory cascade inhibited by local anesthetics. A number of studies have shown that local anesthetics also interfere with migration, priming, and the phagocytic activity of granulocytes [135-138]. Lidocaine has also been proven to reduce the release of pro-inflammatory cytokines, notably IL-1 and TNF-α [139], two of the most important inflammatory mediators. It was also suggested that local anesthetics interfere with the COX pathway, after local anesthetic administration, a reduction in leukotrienes, thromboxane, and prostaglandins is observed [133]. These anti-inflammatory responses also account for a systemic effect of local anesthetics, even when administered regionally. Most of the local anesthetic remains at the site of injection, but a small part undergoes systemic absorption. Animal models have proved that local anesthetics retain their anti-inflammatory properties [140] when administered regionally, even with negligible systemic reabsorption.

Inflammation is not the only component of pain. One of the most important factors in the development of persistent post-surgical pain is the intensity of acute pain [141], thus blocking nociception in the perioperative period could be a valid tool to prevent the emergence of chronic pain [142]. A Cochrane review has shown how RA, most notably epidural analgesia, could help prevent the development of CPSP in one of every four patients after thoracotomy. The results of the review, however, are weakened by the quality of the data from the included studies, thus a consensus has not been reached yet on the role of RA in the development of CPSP [143]. Nevertheless, the preventive role of RA may be surgery- or patient-related, *i.e*. relevant for some specific surgeries and patients only. For example, recent data show no impact of perioperative RA on CPSP [144] after thoracotomy despite improved postoperative analgesia, but better results are reported for breast surgery (especially in patient with catastrophizing habits [145]). Also, RA reduces the incidence of the neuropathic component of pain in this setting (despite no difference in CPSP incidence [146]). In orthopaedic surgery, the preventive role of single-shot techniques is still not supported [147], but benefits may come from the application of continuous techniques that may extend better pain control for up to 1 month [31]. However, the available evidence is probably influenced by heterogeneous methodology and selection bias that excludes patients with a higher risk of CPSP [148].

## CONCLUSION

CPSP is a major issue after surgery, also considering that pain itself is often the indication for surgery, and surgery is expected to improve the patient's quality of life.

Mechanisms underlying CPSP are complex but seem to rely on a dysfunctional adaptation to the painful insult that results in chronic hyperalgesia. In this context, POP remains a main risk factor for CPSP, especially in specific high-risk patients, and effective strategies for POP management may directly (and indirectly) influence CPSP. Unfortunately (to date) a few data exist on preventive strategies for CPSP development, and despite meaningful advancements in POP management they do not always translate into a reduction of CPSP incidence and severity.

However, POP is one item to be considered in a wider model (including biologic, psychologic, and social factors). A dynamic approach to the evolving nature of CPSP is needed (in which pre-existing conditions only partially account for the transition to CPSP). This approach may open a new perspective to face the transitional subacute period after surgery, including the timing of follow-up, identification of objective signs of the evolution to a chronic pain state, and potential targeted and preventive treatments. In this regard, a new approach may reflect why single analgesic strategies have failed (at least in part) to reduce CPSP incidence: “The Holy Grail” in transitional pain medicine does not yet exist, and specific treatments may be more effective when used in different patients (that differently adapt their pain pathways to the surgical insult). As well, the efficacy of any preventative strategy should consider the impact of CPSP on patient’s quality of life, where severity means 1. Intensity; 2. Distress; 3. Function/disability [149]. Any measure of efficacy should include these parameters, which may be defined as the ability to improve patient’s daily activity *i.e*. Eating-Drinking-Mobilizing-Sleeping according to pre-defined goals [150]. Such goals may better define the impact of preventative strategies rather than only measuring CPSP occurrence (yes/no) or severity (as a rude number on a pain scale), as already happened for acute postoperative pain.

## Figures and Tables

**Table 1:** Incidence of chronic post-surgical pain over years and publications for a different type of surgery.

**Type of Surgery**	**Perkins 2000 [** **3** **]**	**Schug 2017 [** **6** **]**	**Correll 2017 [** **8** **]**	**Edgley 2017 [** **9** **]**	**Guimarães-Pereira ** **2017 [** **10** **]**
Limb amputation	60-80%	30-85%	-	-	-
THA	30%	27%	-	-	-
Hysterectomy	5-30%	-	26%	-	-
CS	10%	-	-	-	-
Breast surgery	20-50%	11-57%	30-60%	-	-
Thoracic surgery	25-60%	5-65%	50%	-	-
Sternotomy	20%	7-17%	11%	-	17-37%
Groin hernia	10%	5-63%	10-30% (40)	-	-
TKA	-	13-44%	22%	-	-
Trauma	-	-	-	65%	-

**Abbreviations:** THA: total hip arthroplasty, CS: caesarean section, TKA: total knee arthroplasty.

**Table 2:** Traditional risk factors associated with the development of chronic post-surgical pain (CPSP).

**Predictive Factor**	**Commentary**
Type of surgery	More invasive/long-lasting procedures, with potential risk for nerve damage and high inflammatory reaction [2]
Genetic	Genetic variation may directly affect the nociceptive system, while a genetic signature may also influence other vulnerability factors (mutation in genes involved in the inflammatory process [42-44]) Genetics can also impact on patient’s pain sensitivity and analgesic efficacy/safety of drugs (opioids [45])
Female sex	Females report higher levels of postoperative pain and higher occurrence of CPSP [46]
Younger age	Younger patients are more prone to CPSP. Despite no alterations in pain thresholds and increased risk of neuropathies, older adults have a lower risk [47]
Preoperative anxiety/ Negative psychosocial factors	Anxiety, depression, catastrophizing, and negative attitude are associated with higher levels of postoperative pain and a higher risk for CPSP [37]
Inflammatory state and obesity	The lack of balance between pro- *vs*. anti-inflammatory state promotes CPSP: rheumatologic diseases, fibromyalgia, Irritable bowel Syndrome, Migraine, Raynaud’s, Obesity (adipose tissue displays important pro-inflammatory activity [15, 30, 31, 48])
Pre-existing pain (in the surgical area or not)	Preoperative pain in any site or inflammatory reactions at the site of surgery will predispose to CPSP [5]
Severe or poorly controlled postoperative pain	Intensity and percentage of time spent in severe pain increase the risk to develop CPSP [5]

**Note**: Adapted from Bugada D, Mariano ER. Minerva Anestesiol. 2022; 88(10): 764-767.

## LIST OF ABBREVIATIONS

CFA: Complete Freund’s Adjuvant
CNS: Central Nervous System
COX: Cyclooxygenase
CPSP: Chronic Postoperative Pain
ERAS: Enhanced Recovery After Surgery
LC: Locus Coeruleus
Mg^2+^: Magnesium
NMDA: N-Methyl-D-Aspartate
NSAIDs: Non-steroidal Anti-inflammatory Drugs
POP: Postoperative Pain
QST: Quantitative Sensory Testing
RA: Regional Anesthesia
RVM: Ventromedial Rostral Medulla
SMIR: Skin/Muscle Incision and Retraction
